# *IN VITRO* ANTIMALARIAL ACTIVITY AND TOXICITY STUDIES OF JOHAR (*CASSIA SIAMEA*) LEAVES FROM THREE DIFFERENT LOCATIONS

**DOI:** 10.21010/ajid.v14i2.4

**Published:** 2020-07-31

**Authors:** Ezrani Tasiam, Riesta Primaharinastiti, Wiwied Ekasari

**Affiliations:** Faculty of Pharmacy, Universitas Airlangga, Surabaya 60115, Indonesia

**Keywords:** Anti-malarial, Toxicity, *Cassia siamea*

## Abstract

**Background:**

Antimalarial activity of *Cassia siamea* leaves has been proven by the active compound that has been found, i.e. Cassiarin A. It is known that the quantity of the content of a compound that has the potential as a raw material for medicine can be influenced by various factors including differences in plant origin. This study aims at comparing the antimalarial activity and toxicity of *C.siamea* leaves from three regions with different meters location values above sea level (asl), i.e Pariaman (1,000 m asl), Palu (60 m asl), and Surabaya (2 m asl).

**Materials and Methods:**

The materials used in this study were Johar leaves from Pariaman, Surabaya, and Palu extracted with n-hexane, and 90% ethanol containing 1% tartaric acid. The antimalarial activity test was done with *Plasmodium falciparum* 3D7. The toxicity test applied *MTT ELISA* method.

**Results:**

*C.siamea* leaf that had highest antimalarial activity came from Pariaman with IC_50_ value of 0.006μg/ml, then from Palu was 0.037μg/ml, and the lowest antimalarial activity was from Surabaya that was 0.09μg/ml . In testing the toxicity to get CC_50_, the highest toxicity came from Surabaya with CC_50_ value of 135.81μg/ml, Pariaman with CC_50_ value of 220.82 μg/ml, and the least toxic came from Palu with CC_50_ value of 235.52μg/ml .

**Conclusion:**

*C.siamea* leaf obtained from Pariaman had a selectivity index value that satisfies the requirements of a promising antimalarial effect.

## Introduction

Malaria is one of the deadliest and the most dangerous infectious disease that threatens human lives. To cure and prevent malaria disease, therefore, the government through the Department of Health is continuously striving for an alternative treatment. It is commonly transmitted through the bite of an infected parasite *Plasmodium* female *Anopheles* mosquito. This disease commonly constitutes a threat to the lives of infants, children, and pregnant females.

Numerous attempts have been made by the WHO to prevent the infection of malaria disease, such as recommending administration of the combination treatment with antimalarial drugs (WHO 2015). The combination treatment of antimalarial drugs is taken when a study on the resistance patterns in certain areas have been determined through resistance survey procedure. When a particular drug for certain disease has been resistant for more than 25% of the prevalence, then it is not recommended to be used furthermore. The aims of combination treatment are to improve the efficacy of antimalarial drugs, through antimalarial synergistic activity, and slowing the progression of parasitic resistance to novel antimalarial drugs (WHO 2016).

Indonesia is known as the richest country in natural resources. The one natural resource in this archipelago country includes plants that are beneficial for the needs of human life such as for food, industrial needs, and medicine. One plant that has been known empirically to have benefit as a medicinal plant is *Cassia siamea* (Johar leaf) from Fabacae family that is considered to be a traditional antimalarial medicine.

In spite of its traditional usage, *C.siamea* leaf has been examined to identify the antimalarial activity where *C. siamea* leaf extract and fraction were assessed through *in vitro* studies on *P.falciparum* and *in vivo* in mouse infected with *P.berghei*. It indicated that the alkaloid fraction of the leaf has potential activity. The *in vitro* examination of the fraction produced IC_50_ of 0.24 μg/ml and an isolate Cassiarin A produced IC_50_ of 0.005 μg/ml. The *in vivo* examination in mouse infected with *P. berghei* revealed an ED_50_ of 0.47 mg/kg (Ekasari *et al.*, 2009). An identical study with *C.siamea*, and *C.spetabilis*, undertaken to examine the *in vivo* antimalarial activity of the ethanol extract on mice infected with *P. berghei* gave an ED_50_ of 150 mg/kg (Ekasari 2018). Furthermore, isolation of active compounds from *C.siamea*, alkaloid compounds Cassiari A and B, have been reported (Morita *et al.*, 2007).

The quality of the potential composition of the ingredient used as a raw material can be influenced by various factors including the differences in the native plants, the part of the plant tested, the condition of the plant, and the type of solvent used (Khan *et al.*, 2011). Factors causing differences are internal factors (genetic, ontogeny, and morphogenetic) and external/environmental factors which can be differentiated into two, namely biotic factors (stress due to bacteria, viruses, fungi, and parasites) and abiotic factors (geographical distinction in the place of growth, climate change, type, and condition of soil, water availability, mineral content, and stress due to temperature, radiation, and chemical composition) (Verma and Shukla 2015). Another researcher, Hendrison (2001) reported that differences in altitude will cause different secondary metabolites. In another report by Laily (2012) showed that altitude is one of the factors that influences the growth of a plant. Thus, it is suspected that differences in altitude will affect the growth and components of plants.

In this study, Johar (*C.siamea)* leaf from 3 areas with different location elevations measured as meters above sea level (m asl), namely Pariaman (1000 m asl), Palu (60 m asl), and Surabaya (2 m asl) were subjected to *in vitro* antimalarial activity testing conducted with P.*falciparum* 3D7 culture and the safety was evaluated *in vitro* by *MTT ELISA* method.

## Materials and Methods

The materials used in this study were Johar leaves from three locations: (i). Pariaman which is located in the province of West Sumatera, Indonesia. Pariaman has an altitude of 1000 meters above sea level with a wet tropical climate, an annual rainfall that reaches 368 mm, an average temperature of 26.8°C, and an average humidity of 84.4% (PGAP, 2017). (ii. ) Palu that is located in the province of Central Sulawesi, Indonesia, at an altitude of 60 meters above sea level and with a dry climate, an annual rainfall that reaches 760 mm, an average temperature of 27.7°C, and humidity of 76-80% (SRIP, 2007). (iii). Surabaya which is located in the province of East Java, Indonesia at an altitude of 2 meters above sea level, a rainfall that reaches an average of 172 mm, an average temperature of 30°C, and humidity of 68%-84% (DPBS 2015). The plant was identified by Anshari Maruzi from Systematic Laboratory, with a voucher specimen number YK.01.03/2/2861/2019, and deposited in the Herbarium Tawangmanguense at Medicinal Plant and Traditional Medicine Research and Development Centre, Ministry of Health Republic of Indonesia.

### Extraction and Fractionation

The leaves were dried at room temperature and ground into powder. 10 g of *C.siamea* leaf powder was macerated consecutively using n-hexane solvent (3 times) and ethanol 90% containing 1% tartaric acid (three times) and evaporated at 40oC using rotary evaporator to obtain the crude ethanol extract (Pariaman 1.6 g, Palu 1.8 g and Surabaya 1.1 g). The thick ethanol extract was diluted with distilled water and used for liquid fractionation using 3:1 ethyl acetate (three times) from which crude fraction of ethyl acetate was obtained as follows: Pariaman was 0.89% w/w, Palu was 0.82% w/w and Surabaya 0.89% w/w.

### *In vitro* anti-malarial Activity

*P.falciparum* strain 3D7 (chloroquine-sensitive) was obtained from Institute of Tropical Disease, Universitas Airlangga, Surabaya, Indonesia. The initial parasitemia levels in each well during the experiment of antimalarial activity *in vitro* were 1% parasitemia and 5% hematocrit. One plate consisted of 24 wells (Trager and Jensen, 1976). The suspension parasite (5 ml) was put into a sterile falcone tube 15 ml and then centrifuged at 1,500 rpm for 5 minutes. A total of 4.5 ml of supernatant was removed hence it was estimated that there were around 5% of red blood cells infected with parasites and approximately 50% of hematocrit with a total amount of 500 μL. The parasite suspension was made such that the content of parasitemia obtained was 1% and hematocrit reached 10%, by adding 50% of RBC suspension in a volume of 2000 μL into the tube, then the media was made up to 10 ml.

In addition, the suspension was mixed cautiously using a micropipette until it was well blended. Before being put into a microwell, a thin blood smear was made, the initial parasitemia level was determined at 0 hour before being given test substances. The amount of 500 μL of parasitic cell suspensions was introduced into each well containing 500μL of the test solution. Serial concentrations of the crude fraction of ethyl acetate that were tested for antimalarial activity were 100; 10; 1.0; 0.1; and 0.01μg/ml. Antimalarial assay was done in a 24 microwell plates with 1% initial and experimental parasitemia (1 ml/well of suspension). It was placed in a candle jar and CO_2_ incubator at 37°C for 48 hours. Then, a thin blood smear in a glass slide was made, fixated in methanol, and stained with giemsa 10% for 10 minutes.

The number of parasitemia was observed under a microscope based on 3000’s erythrocytes with 1000 times magnification. The number of parasites at 48 hours incubation was also determined and used for comparison. The percentage of parasite growth was calculated by comparing with the negative control. Then, fifty percent inhibitory concentration (IC_50_) of each extract was determined to express the antimalarial activity. IC_50_ was defined as the concentration of the compound causing 50% inhibition of parasite growth relative to untreated control.

### The Calculation of Inhibition Percentage


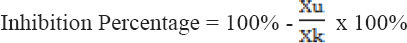


Annotation:

Xu = growth percentage of test solutions

Xk = growth percentage in negative control

### IC_50_ Calculation

The calculation of IC_50_ was done through the use of probit analysis (*unit probability*) by making a correlation curve between probit percent inhibition with logarithm of sample concentration using linear regression of line equations. Table 1 shows how to categorize the antimalarial activity of plant extract based on their IC_50_ values.

**Table 1:** Categorization of plant extract activities towards *P. falciparum* (Kamaraj, 2012)

IC_50_ value (μg/ml)	Category
< 10	Promising
10- 20	Moderate
20 – 40	Good
40- 70	Marginally Potent
>70	Poor

### Toxicity *in vitro* Test using *MTT ELISA* Method

*Huh7it* tissue culture was grown on the media of DMEM in 96 well microplates with the density of 2.3 x 10^4^ cell/well up to 100 μl/well, and the incubation was in CO_2_ 5%, at 37°C for 24 hours. Tissue culture *Huh7it* accrued and stuck to the base of the well microplate. The ethyl acetate fraction of *C.siamea* leaf was diluted with DMSO to achieve a concentration of 10 μg/ml. The tissue culture *Huh7it* media were removed, and 100 μl of the leaf extract fraction was added to the 96 well microplate, and incubated in CO_2_ 5% at 37°C for 48 hours. After this second incubation, the medium was discarded, and 150 μl of *MTT 3-(4,5-dimethyl thiazol-2-yl)-2,5-diphenyl tetrazolium bromide* (10%) solutions was added, followed by incubation in CO_2_ 5% at 37°C for 4 hours. After this incubation, the media were removed and 100 μl of DMSO was added as stop solution, then mixed for one minute. The living cell would interact with *MTT* and changed into purple.

The absorbance was measured at wavelength (λ) of 650 nm and 720 nm with *ELISA* reader, using cell culture *Huh7i*t without the test extract as negative control. Then, the percentage of cell viability was calculated from which CC_50_ value was determined.


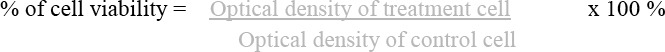


### CC_50_ Calculation

The 50% cytotoxic concentration (CC_50_) was defined as the compounds concentration (μg/ml) required for the reduction of cell viability by 50%, which were calculated by regression analysis.

### Selectivity Index Calculation

Selectivity Index (SI) is defined as the ratio of the cytotoxicity on the human cells to the antimalarial activity. The selectivity index value is obtained as follows.


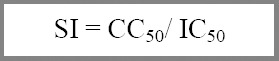


### Data Analysis

IC_50_ values for antimalarial efficacy and CC_50_ for toxicity testing were obtained using probit analysis. To determine a significant difference in the antimalarial effects and the toxicity of *C.siamea* leave extracts from Pariaman, Palu and Surabaya, an ANOVA procedure was used.

## Results

### The Antimalaria Effect of Ethyl Acetate Fraction of C.siamea

From the pulmonary report result of the antimalaria testing and safety evaluation, IC_50_ and CC_50_ values were determined which were then used for calculation of selectivity index.

The ethyl acetate fractions of *C.siamea* from Pariaman, Palu, and Surabaya were used in studying their *in vitro* antimalarial activities. The ethyl acetate fraction was used in this study in accordance with the previous research conducted by Morita *et al*. (2007) and Ekasari (2009) which used ethyl acetate for extraction of active compounds from *C.siamea*, (i.e. alkaloid Cassiari A and B). Those studies reported effective inhibition of malarial parasites by the plant extracts.

The data on percentage of inhibition towards *P.falciparum* as shown in Table 2 were analyzed using the probit analysis. The highest IC_50_ value of the *C.siamea* ethyl acetate faction was from Pariaman with a value of 0.006 μg/ml, Palu was 0.037 μg/ml, and plants from Surabaya showed the lowest antimalarial activity that was 0.09 μg/ml.

**Table 2:** Mean of inhibition and IC_50_ percentage on each partition toward *P.falciparum*

Sample	Consentration (μg/ml)	Inhibition Percentage (%)	IC_50_(μg/ml)
Pariaman	100	98.03	0.006±0.001
	10	88.02	
	1	83.48	
	0.1	78.09	
	0.01	52.99	
Palu	100	88.99	0.037±0.009
	10	80.36	
	1	77.52	
	0.1	64.21	
	0.01	35.38	
Surabaya	100	82.59	0.090±0.063
	10	73.54	
	1	71.29	
	0.1	56.98	
	0.01	27.95	

IC: Inhibitory Concentration

There were significant differences (p < 0.05) between the IC_50_ values of the three samples.

### Toxicity of Ethyl Acetate Fraction of *C.siamea*

The principle of the *MTT* method is the reduction of *MTT* tetrazolium yellow salt by a reductase system. Tetrazolium succinate, when incorporated into the respiration chain in the mitochondria of living cells becomes reduced to form purple formazan crystals which are insoluble in water. The addition of the stopper reagent dissolves this colored crystal and then the absorbance is measured using an *ELISA* reader. The intensity of the purple color that is formed is directly proportional to the number of living cells. Hence, if the intensity of the purple color is greater, it means that the number of living cells is higher (Mossman 1983).

The result from *MTT* assay suggests that the plant extracts have intrinsic biological components that enable them to protect the cells from the damage mediated by *P. falciparum*. Selectivity Index (SI) is used to estimate the potential of molecules or extracts to inhibit parasite growth without toxicity. SI is defined as the ratio of the cytotoxicity on the human cells to the antimalarial activity (Pouplin *et al.*, 2007). Low SI indicates that the antimalarial activity is presumably due to cytotoxic activity of the compound or extract towards the parasite themselves. Meanwhile, higher SI value offers the potential of safer therapy. If the CC_50_>90 μg/ml, the compound is classified as not cytotoxic, if the CC_50_ is between 2 and 89 μg/ml, the compound is classified as moderately cytotoxic, while If the CC_50_<2 μg/ml, the compound is classified as cytotoxic. (Lima 2015).

*In vitro* test of toxicity was conducted by utilizing the *MTT-ELISA* method from the ethyl acetate fraction of Johar leaf (*C.siamea*) taken from Pariaman, Palu, and Surabaya. The viability percentage data of *MTT-ELISA* toxicity test as shown in Table 3 was analyzed by using the probit analysis. The ethyl acetate fraction of *C.siamea* from Surabaya had the highest toxicity with a CC_50_ value of 135.81, then followed by the leaves from Pariaman with CC_50_ value of 220.82, and the least toxicity was from Palu with CC_50_ value of 235.52. From the results of the toxicity test on *C.siamea* ethyl acetate fraction taken from Pariaman, Palu, and Surabaya, it can be asserted that the leaves from different location of plants had different CC_50_. Therefore, there are other compounds with toxic effects in the leaves of *C.siamea*. Qualitative analysis of *C. siamea* had been done for various classes of compounds including alkaloids, tannins, saponins, chromone, flavonoids, carbohydrates, proteins, steroids, terpenoids, cardiac glycosides, and phlobatannins (Lee 2001; Samia, 1978; Usha 2011). It is possible that these toxic effects may be caused by saponins, glycosides, alkaloids such as barakol, inter quinones, and tannins (Wiam, 2005). To find out the content of toxic compounds that play a role in the ethyl acetate fraction of *C.siamea*, further research is needed.

**Table 3:** Percentage of viability and CC_50_ with the *MTT-ELISA* method

Sample	Concentration (μg/ml)	Percentage of viability (%)	CC_50_(μg/ml)
Pariaman	400	9.91	220.82±48.32
	200	60.89	
	100	81.82	
	10	96.13	
	1	99.87	
	0.1	100	
Palu	400	3.35	235.52±12.54
	200	55.97	
	100	90.35	
	10	100	
	1	100	
	0.1	100	
Surabaya	400	1.31	135.81±3.75
	200	22.57	
	78.81	100	
	10	100	
	1	100	
	0.1	100	

CC: Cytotoxicity Concentration

Significant value obtained during the test with the weight fraction equation was (p> 0.05). It further means that there is no significant difference at viability percentage among the three samples.

## Discussion

### Selectivity Index

According to the criteria of antimalarial activity, an extract that gives IC_50_>100 μg/ml is considered inactive while an extract that gives IC_50_<100 μg/ml can be considered as having a potential as an antimalarial. *In vitro* screening to determine *P.falciparum* inhibition activity of plant extracts with IC_50_ value less than 10 μg/ml provides a source for the development of new antimalarial drugs. To obtain risk-free antimalarial treatment, cytotoxicity assay is carried out to obtain SI value. Based on SI value as parameter of pharmacological effect, the extract with SI<4 can be classified as marginally active, SI 4-10 classified as partially active, and SI>10 classified as active antimalarial. Accordingly, as indicated in Table 4, the leaf extracts from the three locations tested had satisfactory selectivity index (>10), indicating satisfactory therapeutic potentials. The presence of bioactive compounds in herbal extracts provides possibilities for a wide range of therapeutic uses. (Weniger 2001).

**Table 4:** Antimalarial Selectivity Index values of plant extracts from the three locations.

Sample	IC_50_	CC_50_	SI
Pariaman	0.006	220.82	36803
Palu	0.037	235.52	6365
Surabaya	0.09	135.81	1509

IC: Inhibitory Concentration CC: Cytotoxicity Concentration SI: Selectivity Index

Thus, from results of the antimalarial activities of the ethyl acetate fraction of *C.siamea* taken from Pariaman, Palu, and Surabaya, it is apparent that different locations resulted in different IC_50_ values for the plant extracts. These differences are due to differences in the metabolite profile and compound constituents (Hendrison 2001). Therefore, it is suggested that there are differences in the levels of the active compounds in *C.siamea* responsible for antimalarial activity.

Selectivity Index value plays a role as a parameter to determine whether or not an extract with antimalarial activity is risk-free. The value of Selectivity Index of an extract that has antimalarial activity is applied in determining whether the extract can be developed further as an antimalarial for pharmacological usage.

Altitude generates changes in temperature and climate conditions. The study of metabolite profiles of *C.roseus* from diverse regions with different altitudes resulted in differences in the metabolite content of phenolic compounds; resulting in differences of antioxidant activity. This further signifies that geographical conditions affect metabolites (Verma and Shukla 2015). A study by (Figueiredo 2008), reports that the secondary metabolite content in plants is influenced by a great deal of factors, including genetic, environmental (biotic and abiotic) factors, physiological conditions, geographical variations and, evolution. Differences in location based on altitude and geographical conditions are expected to affect plant growth and development. As a result, the process of metabolism in these plants will also be affected hence, the compounds produced will be different at each altitude. Metabolites are classified into two: primary and secondary metabolites. Primary metabolites that are formed in limited quantities constitute important factors for the growth and life of organism. Secondary metabolites are not used by plants for growth and are generated more when plants are in stressful conditions, as stated by (Dicosmo, 1984). Secondary metabolite compounds are chemical compounds that generally have bioactivity and can also function as a protection to plants from pests and diseases or from the environment.

The production of secondary metabolites is certainly affected by several factors (Dicosmo 1984) such as light, pH, aeration and certain microorganisms that affect the production of secondary metabolite compounds. Hence, in different altitudes where the height of the place also affects the ambient temperature, the biochemical processes taking place in the plants are affected. Based on the results of this study, it is apparent that differences in the geographical locations of the plants and in altitudes above sea level result in significant differencies in their antimalarial activities and toxicities. The ethyl acetate fraction of *C.siamea* from Pariaman had the highest antimalarial activity, whereas the ethyl acetate fraction of *C.siamea* from Surabaya had the highest toxicity . This shows that the active compound of *C.siamea* which plays a role in antimalarial activity has no relationship with the toxicity profile of the plant. Thus, further research is needed to determine the levels of active compounds from *C.siamea* ethyl acetate fraction from various regions to get the highest level as a raw material of antimalarial drugs.

## Conclusion

This study reports that the Johar (*C.siamea*) leaves obtained from Pariaman yielded satisfactory selectivity index value which meets the requirements for further evaluation for pharmacological effects. Accordingly, the results indicate that the leaves can be developed as a raw material for antimalarial treatment. This study further affirms that the location of *C.siamea* in terms of its altitude affects the antimalarial activity and toxicity of ethyl acetate fraction of the leaves.

**List of Abbreviations:**
*C.roseus*: - *Catharanthus roseus*
*C.siamea*: – *Cassia siamea*
CC_50_: - Cytotoxicity Concentration
DMEM: - Dulbecco’s Modified Eagle Medium
DMSO: – Dimethyl sulfoxide
ED_50_: – Effective Dose
ELISA: - Enzyme-linked Immunosorbent Assay
*Huh7it*: – *devirat human hepatocarcinoma*
IC_50_: – Inhibitory Concentration
LD_50_: - Lethal Dose
m asl: – Meters Above Sea Level
*MTT*: - 3-(4,5-*dimethyl thiazol-2-yl*)-2,5-*diphenyl tetrazolium bromide*
*P. falciparum*: – *Plasmodium falciparum*
RBC: – Red Blood Cell
SI: – Selectivity Index
*3D7*: – *plasmodium falciparum strain chloroquine sensitive*
